# Semi-Automatic Refinement of Myocardial Segmentations for Better LVNC Detection

**DOI:** 10.3390/jcm14010271

**Published:** 2025-01-06

**Authors:** Jaime Rafael Barón, Gregorio Bernabé, Pilar González-Férez, José Manuel García, Guillem Casas, Josefa González-Carrillo

**Affiliations:** 1 Computer Engineering Department, University of Murcia, 30100 Murcia, Spain; jrafael.baron@um.es (J.R.B.); pilargf@um.es (P.G.-F.); jmgarcia@um.es (J.M.G.); 2 Hospital Universitari Vall d’Hbron, 08035 Barcelona, Spain; gcasasmasnou@gmail.com; 3 Hospital Virgen de la Arrixaca, 30120 Murcia, Spain; josegonca.alarcon@gmail.com

**Keywords:** left ventricular non-compaction diagnosis, cardiomyopathies, convolutional neural networks, MRI Image segmentation

## Abstract

**Background:** Accurate segmentation of the left ventricular myocardium in cardiac MRI is essential for developing reliable deep learning models to diagnose left ventricular non-compaction cardiomyopathy (LVNC). This work focuses on improving the segmentation database used to train these models, enhancing the quality of myocardial segmentation for more precise model training. **Methods:** We present a semi-automatic framework that refines segmentations through three fundamental approaches: (1) combining neural network outputs with expert-driven corrections, (2) implementing a blob-selection method to correct segmentation errors and neural network hallucinations, and (3) employing a cross-validation process using the baseline U-Net model. **Results:** Applied to datasets from three hospitals, these methods demonstrate improved segmentation accuracy, with the blob-selection technique boosting the Dice coefficient for the Trabecular Zone by up to 0.06 in certain populations. **Conclusions:** Our approach enhances the dataset’s quality, providing a more robust foundation for future LVNC diagnostic models.

## 1. Introduction

### 1.1. Clinical Context of LVNC

Cardiovascular diseases remain the leading cause of mortality worldwide, accounting for approximately 32% of all deaths globally [1,2]. Early and accurate detection of cardiac anomalies is crucial for improving patient outcomes. Among these illnesses, left ventricular non-compaction cardiomyopathy (LVNC) is a severe but rare cardiac disorder characterized by excessive trabeculations and deep recesses in the left ventricular myocardium due to incomplete myocardial compaction during embryonic development. Given the lack of morphometric evidence for ventricular compaction in humans, the ESC recommends the term ‘hypertrabeculation’ instead of LVNC, particularly when the condition is transient or clearly of adult onset [3]. LVNC can cause a spectrum of symptoms ranging from fatigue and dyspnea to heart failure, and it is also associated with an increased risk of arrhythmias and thromboembolic events. Hypertrabeculation in hypertrophic cardiomyopathy presents additional diagnostic difficulties due to the asymmetry of wall thickness in the different areas of the left ventricular myocardium. An association has been found between the presence of crypts (trabeculae) and different mutations of sarcomeric genes in genetically affected patients with mild forms of hypertrophy. This suggests that quantification of trabeculae in hypertrophic cardiomyopathy may add diagnostic or prognostic value in these patients [4,5].

Magnetic resonance imaging (MRI) is the preferred modality for diagnosing LVNC, providing high-resolution images that allow detailed visualization of myocardial structures. Quantifying the degree of trabeculation is essential for diagnosing LVNC [6,7,8,9] and assessing its severity, typically achieved by calculating the percentage of trabeculated myocardial volume (see Section 1.4).

### 1.2. Evolution of Segmentation Methods

The accurate segmentation of the left ventricular myocardium is crucial for the reliable quantification of trabeculated myocardial volume. Manual segmentation methods are precise; however, they are labor-intensive, time-consuming, and susceptible to inter-observer variability. Consequently, there has been significant interest in developing automated segmentation techniques utilizing computer vision algorithms. In an initial research study, we proposed QLTVHC [10], a semi-automated software tool leveraging medical expertise. QLTVHC was developed to quantify the extent of non-compaction using cardiac MRI. This tool employed conventional computer vision techniques and required cardiologists to adjust it manually. Afterwards, in order to improve QLTVHC, we proposed an automatic tool called SOST [11] by applying a least squares adjustment.

Another way to automatically segment images is to use deep neural networks (DNNs). Due to their ability to learn complex patterns directly from data, DNNs have been successfully applied across various scientific domains, including medical imaging [12,13,14,15]. DNNs are considered foundational tools in modern artificial intelligence (AI) [16]. Consequently, our subsequent step was to explore this approach by proposing DL-LVTQ [17]. DL-LVTQ is an automated method based on a U-Net architecture designed specifically for the diagnosis of LVNC [17]. Using the fully automatic segmentation method of DL-LVTQ [17], we showed that the U-Net model can accurately segment the left ventricle to detect LVNC [18]. However, the segmentations obtained, while promising, were not flawless, limiting our ability to optimize the performance of our models.

In addition, to improve segmentation accuracy in myocardial images, we increased the dataset size and tested more advanced neural network architectures such as MSA-UNet, AttUNet, and U-Net++ [18]. We also implemented a clustering algorithm to reduce hallucinations [18]. However, our best results reached a Dice score of 0.87 in the Trabecular Zone on a particular subset of the dataset (P).

### 1.3. Proposed Approach and Objectives

Our previous work showed us the potential of semi-automatic segmentation methods in improving both the diagnosis of LVNC and the segmentation images used for training these models. Due to the complexity of left ventricular trabeculations and the limitations of fully automatic methods for creating training datasets, we believe that an approach that combines the strengths of automatic segmentation with expert validation can improve accuracy and efficiency.

Consequently, in this current study, we propose a new semi-automatic method to further improve the quality of these segmentations. This new method combines user-friendly interfaces with predictions from our existing neural network models. This method allows for the refinement of segmentations with minimal manual intervention, achieving an optimal balance between efficiency and accuracy.

The main aim of this paper is to enhance the accuracy and reliability of myocardial image segmentation by improving the quality of the training datasets used for deep learning models. We design and implement a semi-automatic image correction framework that refines and corrects segmentations within the training data. This framework tackles common issues such as incomplete border coloring, hallucinated structures, and general inconsistencies by combining multiple neural network outputs with user intervention. To achieve this, this work comprehends:

1. A Semi-Automatic Image Fixing Method: We create a cross-validation framework that utilizes multiple neural network models to identify and correct errors in image segmentation. Our aim is to propose a refined version for each image by identifying and automatically correcting the areas of disagreement between the outputs of multiple trained models. We manually validate and incorporate these corrections to establish the blob-selection method.
2. An Image Fixer Program: We design and implement a user-friendly software tool for interactive image segmentation correction. This tool displays the original segmentation alongside the corrected version, highlighting differences and enabling clinical experts to apply targeted adjustments efficiently. We refer to this process as the manually fixed method.
3. An Image Selector Tool: We develop an application that presents multiple segmentation options for a given image and allows users to select the most accurate one. This tool facilitates subjective evaluation and selection and ensures that the final segmentation used for diagnosis is the one with the highest quality.

Through these initiatives, we seek to refine existing segmentation methods by leveraging automated correction techniques and expert validation. By improving the training dataset to better reflect the true myocardial segmentation, we develop more reliable models for the LVNC diagnosis and contribute to better patient outcomes.

### 1.4. Left Ventricular Non-Compaction Cardiomyopathy (LVNC)

LVNC is diagnosed by quantifying the degree of trabeculation, which is essential for even assessing its severity. To calculate the percentage of trabeculated myocardial volume, we use the equation:

(1) $$VT\%=\frac{TZ}{TZ+EL}\cdot 100\;$$

where *TZ* represents the volume of the trabeculated zone and *EL* denotes the volume of the compact external layer. A threshold of 27.4% is commonly used as a diagnostic criterion [6], with values above this indicating significant hypertrabeculation consistent with LVNC.

### 1.5. Semi-Automatic Segmentation Methods

Semi-automatic segmentation methods have gained widespread use in medical imaging. For example, a semi-automatic approach to facial wrinkle detection reduces manual effort while improving detection accuracy by focusing on intricate textures [19]. In bladder cancer diagnosis, semi-automatic segmentation achieves results comparable to manual methods, significantly reducing processing time [20]. Similarly, in dental imaging, a semi-automatic coarse-to-fine segmentation method utilizing intuitive single clicks effectively segments complex 3D tooth models, improving efficiency and accuracy over fully automatic approaches [21]. The use of these methods in rectal cancer tumor segmentation further highlights the growing preference for semi-automatic approaches, which streamline workflows without compromising precision [22].

## 2. Materials and Methods

We developed a semi-automatic correction method to address segmentation errors in a database of images, including issues such as incomplete border coloring, hallucinated trabeculae in empty regions, and general inconsistencies.

Although the images generated from the proposed method here are automatic, we call it semi-automatic since many of the changes provided by these methods are not desired and should, therefore, be sorted out. For this reason, we developed a user-friendly application to refine and compare these segmentations efficiently.

### 2.1. Cross-Validation Method

**Figure 1:**
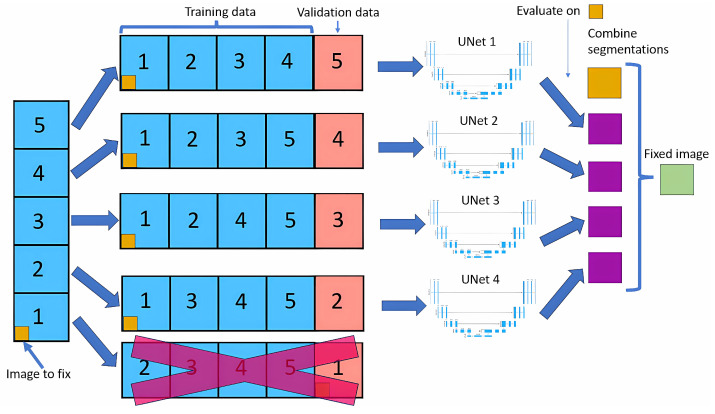
Cross-validation method.

The cross-validation method splits the dataset into five equal parts (called folds). For each fold, one part is used as the validation set, while the remaining four are used to train the neural network model. Figure 1 shows this method. This process is repeated five times, with each part used as the validation set precisely once, resulting in a total of five neural nets being trained.

For each image, we look at the four outputs of neural nets that have used that image in the training dataset. For each pixel of that image, we look at the four outputs, and if three of them contain the same output class, that will be the new value for that pixel; if not, the value will remain the same as the original.

Modifying only images in the training dataset allows the model to learn that image in depth, making it unlikely to do any big changes, leading to more stable results than if we did a normal cross-validation.

**Figure 2:**
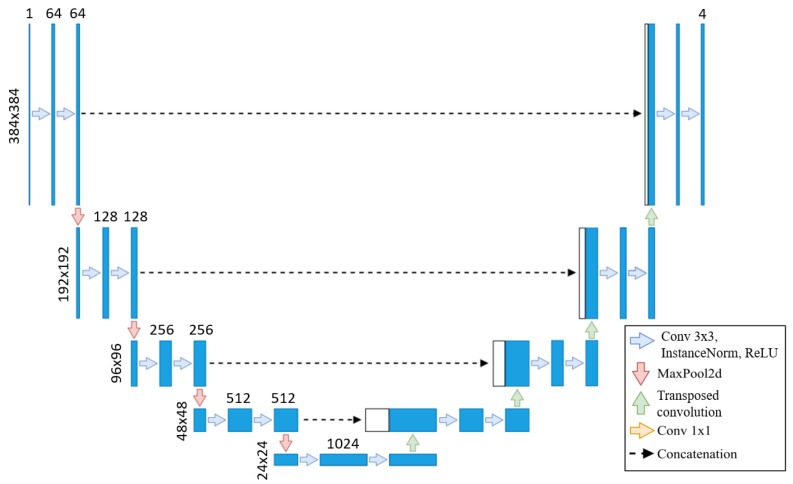
U-Net from [17] adapted to our use case.

We use the baseline U-Net in this method due to the lengthy training times of neural networks, with the same architecture applied across all training sets, as shown in Figure 2. As the original segmentations were made at 800 × 800, we upscale our images to 800 × 800 but then cropped them to 384 × 384 to focus on the left ventricle. Our U-Net takes these 384 × 384 images as input, as the images are crop to focus on the left ventricle, eliminating the need for additional downsampling. Each step of the encoder path includes two 3 × 3 convolutions with instance normalization and leaky ReLU (0.01 negative slope), followed by a stride-two convolution to reduce spatial dimensions. The resulting network has different filters in each convolutional block, ranging from 64 in the first layer to 1024 in the bottom layer. The U-Net then outputs a segmentation map with the same 384 × 384 resolution, with four channels representing each class’s probabilities.

This automated framework minimizes the need for manual segmentation adjustments by offering modification suggestions to clinical experts, enabling them to identify patterns that might otherwise go unnoticed.

### 2.2. Neural Nets Used for Testing

After fixing the images, we perform cross-validation with U-Net on train/val/test sets. We train on full-sized 800 × 800 images to allow a fair comparison to the U-Net model from our previous work [18]. We adopt the same architecture as the prior study to handle these larger images effectively, incorporating additional layers designed to process 800 × 800 inputs. This ensures both consistency in architecture and the capacity to manage the increased resolution. First, images are downsampled to 200 × 200 using two 3 × 3 convolutions with instance normalization and leaky ReLU activations (negative slope 0.01), each with a stride of two, effectively halving each dimension. The model then processes this 200 × 200 input and outputs at the same resolution.

To upscale back to 800 × 800 before the final output, we add a decoding layer comprising two transposed convolutions (stride of two), each followed by two 3 × 3 convolutions with instance normalization and leaky ReLU. This configuration restores the spatial resolution to 800 × 800, preparing the model for the final segmentation output.

### 2.3. Image Fixer

Now, the goal is to obtain a segmentation for our image that is closest to what we believe to be as correct as possible. To facilitate this, we use two different segmentation methods.

**Figure 3:**
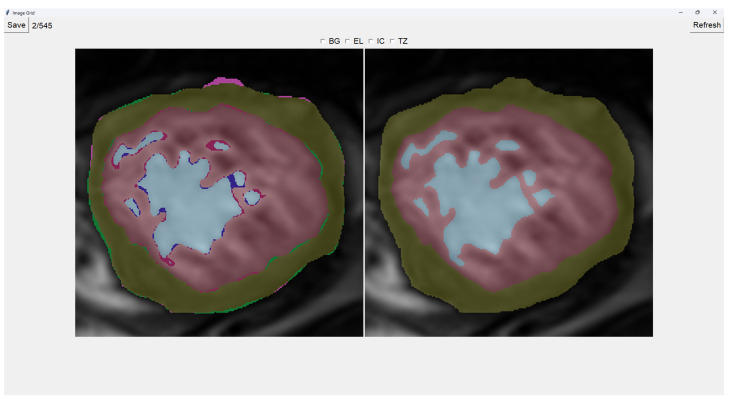
Initial interface for Image fixer.

This program presents two images as shown in Figure 3. The image on the left is obtained from our previous method (QLVTHC), while the one on the right is obtained from our neural networks (Section 2.1). The final output of our program is the segmentation on the right.

We use a muted color scheme to make the colors distinguishable for colorblind people. In the image on the right, the External Layer is olive green, the Internal Cavity is cyan, and the Trabecular Zone is rose.

On the figure on the left, we mark the differences between both segmentations. This way, where there is an External Layer on the left and something else on the right, we mark it with green. For additional Internal Cavity, we mark it with blue; for Trabeculae, we mark it with wine red; for Background, we mark it with purple.

The differences between both images can be leveraged for easy transformations of the output image. The transformation is applied to the image on the right by simply clicking one of the differences. For example, if we click a blob that is colored green on the left (meaning an additional External Layer), an External Layer will appear in that zone of the image on the right. Some of these blobs are very small, so you can select them by right-clicking and dragging over them for ease of use.

**Figure 4:**
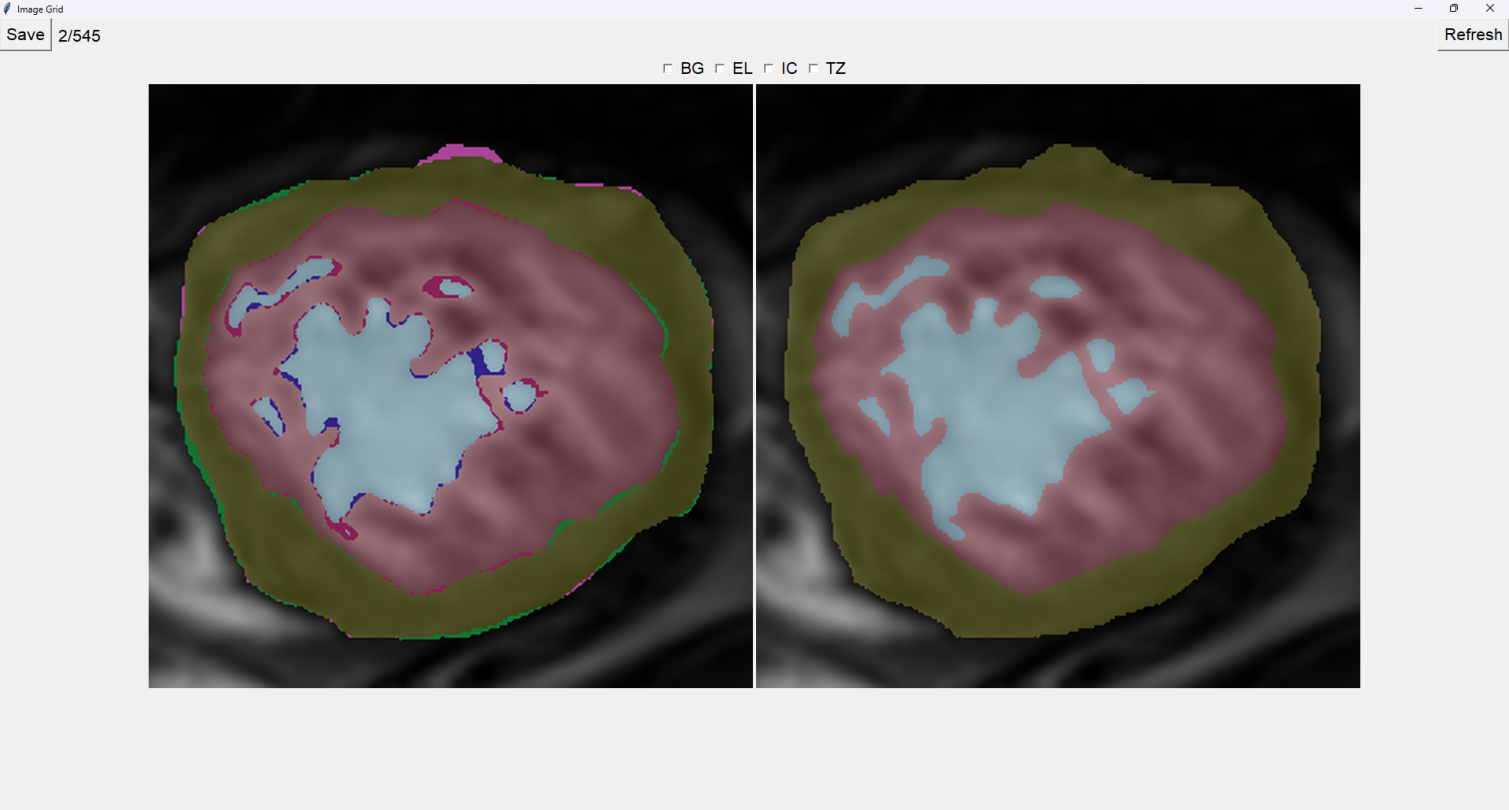
Painting Trabecular Zone on output image.

Painting directly on the output image (the image on the right) is also possible. For this, we select either BG (Background), EL (External Layer), IC (Internal Cavity), or TZ (Trabecular Zone). Then, we simply left-click where we want to paint (as shown in Figure 4).

When we finish the image, we just save the image and go to the next image automatically. However, if we want to restart the editing of the image, we can refresh it and erase the modifications made.

Finally, we can also toggle the segmentation which helps us view the borders’ coloring. It is important to note that you can still paint when the image is toggled to the raw image.

### 2.4. Image Selector

This tool allows the user to select the most accurate segmentation for a given image from three different segmentations. The process is designed as a blind test to ensure an unbiased evaluation, allowing the user to choose the segmentation that most closely represents reality.

**Figure 5:**
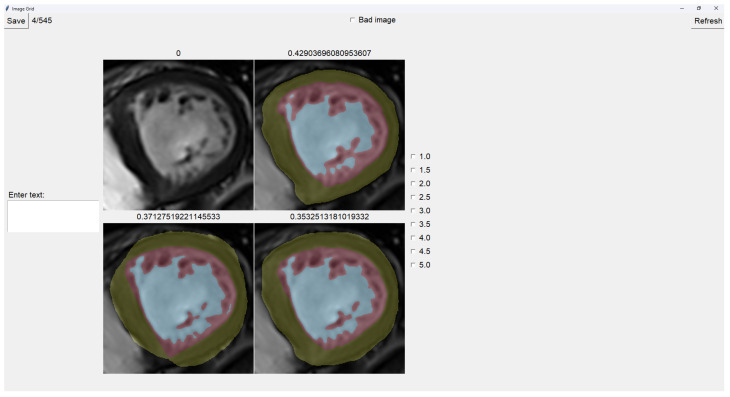
Image selector interface.

The program presents four images on a grid, as shown in Figure 5. The raw image is presented in the top-left corner, while the other three images are segmentations obtained differently. These three images are randomly placed in each iteration to ensure that this is a blind test.

On top of each image, we show the percentage of image trabeculation for the given segmentation (Equation (1)).

Again, we use a muted color scheme to make the colors distinguishable for colorblind people. The External Layer is olive, the Internal Cavity is cyan, and the Trabecular Zone is rose.

**Figure 6:**
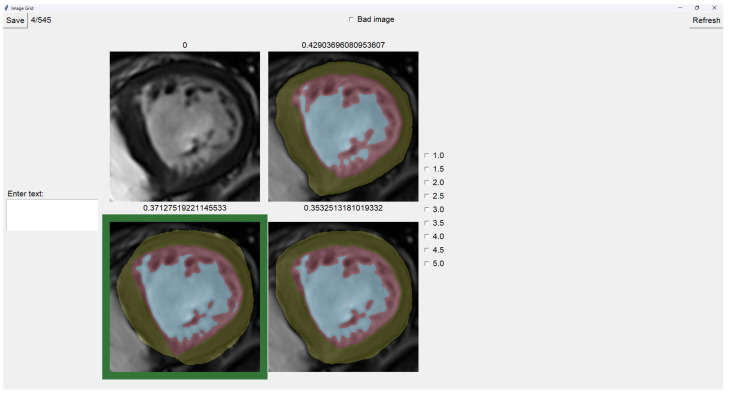
Image selector with the bottom-left image selected.

To select an image, you have to left-click on the image, and then a green box appears around the selected image (see Figure 6). On the right, we can choose a mark of 1–5, indicating how good the image is. Table 1 shows the subjective evaluation scale proposed in [23]. Finally, we can save it, which automatically brings up the next batch.

In addition, we can give feedback in the textbox on the left. We can also indicate whether the quality of the image is bad by selecting the checkbox at the center top.

It is possible to zoom in on an image using the mouse wheel. It is also possible to toggle between viewing each image as the raw image for easy comparison between borders.

The three images presented (see Figure 5 and Figure 6) are obtained using the following methods:

1. Original targets: QLVTHC output [11].
2. Blob-selection method: We compare the cross-validation method (Section 2.1) for improving images to the QLVTHC method on the image fixer. We improve the images only by choosing the best blobs based on their differences.
3. Manually fixed method: We manually fix 100 images by coloring on the image fixer (left-click). We perform the cross-validation method on these 100 fixed images and continue manually fixing 400 more images using these outputs as a new base.

### 2.5. Datasets

The datasets used in this study are derived from three hospitals: Virgen de la Arrixaca of Murcia (HVAM), Mesa del Castillo of Murcia (HMCM), and Universitari Vall d’Hebron of Barcelona (HUVHB). These hospitals provide the medical imaging data for the analysis and contribute a variety of patient profiles that enrich the study.

HVAM operates two scanners, one from Philips and one from General Electric, with a field strength of 1.5 T. The acquisition matrices for these scanners are 256 × 256 pixels and 224 × 224 pixels, respectively, with pixel spacing of 1.5 × 1.5 × 0.8 mm and 1.75 × 1.75 × 0.8 mm. HMCM uses a General Electric model scanner identical to HVAM’s, while HUVHB utilizes a 1.5 T Siemens Avanto scanner with an acquisition matrix of 224 × 224 pixels. For all institutions, the images were captured using balanced steady-state free precision (b-SSFP) sequences. The primary parameters for the scans, including echo time (1.7 ms), flip angle (60º), slice thickness (8 mm), slice gap (2 mm), and 20 imaging phases, were consistent across all hospitals. All patients underwent the scans while in apnea, synchronized with ECG, and without using contrast agents.

The original dataset comprises data from three subsets: P, X, and H. Set P, from HVAM, consists of 293 patients (2381 slices) with hypertrophic cardiomyopathy (HCM). Set X, from HMCM, includes 58 patients (467 slices) with various heart conditions, including HCM. Finally, set H, from HUVHB, comprises 28 patients (196 slices) diagnosed with left ventricular non-compaction cardiomyopathy (LVNC) according to the Petersen criteria.

Given the time-intensive nature of manual segmentation methods, we use a representative subset of 545 modified segmentations from the original dataset. This subset includes 355 slices from the P dataset, 75 from the X dataset, and 115 from the H dataset. The selection process ensures that the larger dataset’s diversity of heart conditions and image characteristics is still adequately represented in the smaller subset.

To enable comparison with the results of our previous U-Net model, we train our new models using a train/validation/test split. From our dataset of 545 images, we retain the 113 images used as a test in our prior work [18]. The remaining 432 images were divided into 5 non-overlapping folds, ensuring no patient data were shared between folds, allowing us to create a cross-validation dataset. For the creation of these training folds, 297 images were from P, 53 from X, and 82 from H, while for testing, 58 were from P, 22 from X, and 33 were from H.

## 3. Results

This section presents the results obtained by applying the blob-selection and manual correction methods to the segmentation tasks. First, we compare the performance of these methods against the original segmentation targets.

### 3.1. Comparison Between Models Generated by the Datasets

To determine whether modifying the dataset has led to improvements in the test Dice scores, we evaluated the cross-validation U-Nets from [18] and compared them to cross-validation U-Nets trained on the adjusted datasets. Specifically, we trained five U-Nets for the blob-selection and manually fixed methods, evaluating each model on test images from their respective datasets. The results of these evaluations are shown in Table 2, Table 3 and Table 4.

### 3.2. Dataset Comparison with Original Segmentation Targets

We evaluate the performance of both the blob-selection and manual correction methods by comparing their resulting segmentations with the original segmentation targets using the Dice coefficient. Table 5 and Table 6 show the average Dice coefficients for each method across the three populations: P, X, and H.

### 3.3. Comparison Between Blob-Selection and Manual Correction Methods

To assess how closely the blob-selection method approximates the manual corrections, we directly compare the segmentations from both methods, as shown in Table 7, with the corresponding Dice coefficients from this comparison.

## 4. Discussion

As shown in Table 2, the baseline U-Net model achieves Dice coefficients ranging from 0.82 to 0.87 for the Trabecular Zone. In comparison, the blob-selection and manually fixed methods (Table 3 and Table 4) exhibit improved Dice coefficients, with increases of up to 0.06 for the H population. This improvement is particularly noteworthy given that the baseline model was trained with seven times more data per neural network than the adjusted models. Despite having less training data, the blob-selection and manual methods yield higher accuracy and stability, especially in regions like the Trabecular Zone. For the P population, both methods achieve a 0.02 increase in Dice coefficients. However, for the X Trabecular Zone, no improvement is observed, potentially due to the lower representation of X images within the 432 images used in the training sets.

The blob-selection method consistently outperforms both the baseline and the manually fixed method, though the difference with the latter is slight. This is because the blob-selection method only allows changes proposed by the cross-validation method, which tends to homogenize results. While this approach reduces variability, it may also exclude changes that would increase accuracy by correctly capturing variations.

Modifying an image using the blob-selection method takes approximately 20 to 30 s, while manual correction requires around 2 to 3 min per image. This significant difference indicates that the blob-selection method is much more efficient in terms of time.

Table 5 shows that the blob-selection method achieves high Dice coefficients across all populations, with average values around 0.94. In contrast, the manual correction method yields lower Dice coefficients, as shown in Table 6, with average values ranging from 0.80 to 0.89. The higher Dice coefficients for the blob-selection method indicate a closer similarity to the original segmentation targets. This is expected because the blob-selection method makes more conservative adjustments, while the manual correction method allows for more significant modifications that may deviate further from the original targets.

The results in Table 7 indicate a slight improvement in Dice coefficients for the blob-selection method compared to those of the manual correction method and the original targets (Table 6). This slight increase of approximately 0.03 suggests that the blob-selection method aligns segmentations closer to the desired outcomes. However, the difference may not be substantial enough to fully correct the images on its own.

Given the balance between accuracy and efficiency, a mixed-method approach could benefit larger datasets. Initially, a subset of images could be manually corrected to create a robust foundational model, enhancing cross-validation predictions. Subsequently, the remaining images could primarily utilize the blob-selection method, with manual adjustments as necessary. This approach not only ensures data quality and optimizes resource allocation but also leverages the strengths of both methods: the automatic blob-selection method provides a rapid and reliable baseline, while expert manual adjustments further refine the dataset, maintaining both high accuracy and flexibility for future modifications.

## 5. Conclusions

This paper enhances the approach to diagnosing left ventricular non-compaction cardiomyopathy (LVNC) by introducing a semi-automatic framework designed to improve the quality of segmentations in training datasets, ultimately yielding more robust models. Utilizing cross-validation with multiple neural networks, the framework includes tools such as the Image Fixer and Image Selector to refine segmentation quality within the training dataset. This approach addresses a primary bottleneck in model effectiveness: the quality of input segmentations.

Our results demonstrate that improving segmentation quality in the training data substantially impacts the effectiveness of neural network models. Notably, despite being trained on datasets with seven times fewer images per neural network than the baseline model, the models trained on adjusted datasets using the blob-selection and manual correction methods achieved superior performance.

The fast image modification method (blob-selection method) led to an alignment improvement of approximately 0.03 in the average Dice coefficient. This leaves significant room for improvement, making it necessary to first create good segmentations via the proposed manual method.

We intend to provide clinicians with the Image Fixer tool, allowing them to refine the automatically generated segmentation immediately after receiving it, as needed. This could potentially improve both the efficiency and accuracy of future model training datasets. Once we apply these methods to all the images, we anticipate a significantly improved model, as previous papers have shown that more data leads to better, more robust models.

## Figures and Tables

**Table 1:** Subjective evaluation scale proposed in [23].

Score	Segmentation Quality
5.0	Exact match: there are no noticeable differences
4.5	
4.0	Noticeable differences: they are not diagnostically significant
3.5	
3.0	Small diagnostically significant differences
2.5	
2.0	Significant diagnostic information is lost
1.5	
1.0	Large diagnostically significant differences

**Table 2:** Dice coefficients from the U-Net in [18] on its images from our test set.

Population	Dice EL	Dice IC	Dice TZ	Average Dice
P	0.90±0.02	0.963±0.002	0.87±0.01	0.91±0.01
X	0.88±0.02	0.967±0.003	0.87±0.003	0.90±0.01
H	0.89±0.01	0.937±0.003	0.82±0.01	0.88±0.01

**Table 3:** Dice coefficients from the U-Net trained on blob-selection images on its test set.

Population	EL	IC	TZ	Media
P	0.928 ± 0.004	0.97 ± 0.01	0.895 ± 0.004	0.930 ± 0.004
X	0.90 ± 0.01	0.97 ± 0.01	0.87 ± 0.01	0.91 ± 0.01
H	0.90 ± 0.01	0.95 ± 0.01	0.87 ± 0.02	0.91 ± 0.01

**Table 4:** Dice coefficients from the U-Net trained on manually fixed images on its test set.

Population	EL	IC	TZ	Media
P	0.91 ± 0.01	0.966 ± 0.004	0.89 ± 0.02	0.92 ± 0.01
X	0.88 ± 0.02	0.97 ± 0.01	0.87 ± 0.02	0.90 ± 0.01
H	0.87 ± 0.02	0.93 ± 0.01	0.88 ± 0.02	0.89 ± 0.02

**Table 5:** Dice coefficients comparing original segmentation targets with those obtained using the blob-selection method.

Population	Dice EL	Dice IC	Dice TZ	Average Dice
P	0.94±0.02	0.98±0.02	0.91±0.04	0.94±0.04
X	0.93±0.02	0.98±0.01	0.90±0.03	0.94±0.03
H	0.93±0.02	0.95±0.03	0.86±0.05	0.92±0.05

**Table 6:** Dice coefficients comparing original segmentation targets with those obtained through manual correction.

Population	Dice EL	Dice IC	Dice TZ	Average Dice
P	0.89±0.03	0.94±0.03	0.85±0.05	0.89±0.03
X	0.84±0.05	0.95±0.02	0.82±0.05	0.87±0.04
H	0.85±0.04	0.88±0.05	0.75±0.05	0.80±0.10

**Table 7:** Dice coefficients comparing the blob-selection method with the manual correction method.

Population	Dice EL	Dice IC	Dice TZ	Average Dice
P	0.91±0.03	0.95±0.02	0.87±0.02	0.90±0.03
X	0.88±0.04	0.96±0.02	0.85±0.02	0.90±0.03
H	0.87±0.03	0.90±0.05	0.80±0.06	0.84±0.08

## Data Availability

The dataset used to support the findings of this study was approved by the local ethics committee and so cannot be made freely available. Requests for access to these data should be made to the corresponding author, Gregorio Bernabé, gbernabe@um.es.
